# The role of nicotinic acetylcholine receptors in autosomal dominant nocturnal frontal lobe epilepsy

**DOI:** 10.3389/fphys.2015.00022

**Published:** 2015-02-11

**Authors:** Andrea Becchetti, Patrizia Aracri, Simone Meneghini, Simone Brusco, Alida Amadeo

**Affiliations:** ^1^Department of Biotechnology and Biosciences and NeuroMi-Milan Center for Neuroscience, University of Milano-BicoccaMilano, Italy; ^2^Department of Biosciences, University of MilanoMilano, Italy

**Keywords:** ADNFLE, CHRNA2, CHRNA4, CHRNB2, GABA, nAChR, prefrontal cortex, sleep-related epilepsy

## Abstract

Autosomal dominant nocturnal frontal lobe epilepsy (ADNFLE) is a focal epilepsy with attacks typically arising in the frontal lobe during non-rapid eye movement (NREM) sleep. It is characterized by clusters of complex and stereotyped hypermotor seizures, frequently accompanied by sudden arousals. Cognitive and psychiatric symptoms may be also observed. Approximately 12% of the ADNFLE families carry mutations on genes coding for subunits of the heteromeric neuronal nicotinic receptors (nAChRs). This is consistent with the widespread expression of these receptors, particularly the α4β2^*^ subtype, in the neocortex and thalamus. However, understanding how mutant nAChRs lead to partial frontal epilepsy is far from being straightforward because of the complexity of the cholinergic regulation in both developing and mature brains. The relation with the sleep-waking cycle must be also explained. We discuss some possible pathogenetic mechanisms in the light of recent advances about the nAChR role in prefrontal regions as well as the studies carried out in murine models of ADNFLE. Functional evidence points to alterations in prefrontal GABA release, and the synaptic unbalance probably arises during the cortical circuit maturation. Although most of the available functional evidence concerns mutations on nAChR subunit genes, other genes have been recently implicated in the disease, such as *KCNT1* (coding for a Na^+^-dependent K^+^ channel), *DEPD5* (Disheveled, Egl-10 and Pleckstrin Domain-containing protein 5), and *CRH* (Corticotropin-Releasing Hormone). Overall, the uncertainties about both the etiology and the pathogenesis of ADNFLE point to the current gaps in our knowledge the regulation of neuronal networks in the cerebral cortex.

## Introduction

Sleep has been long known to facilitate the occurrence of epileptic seizures, particularly during non-rapid eye movement (NREM) sleep (Boursoulian et al., 2013). Among the most common epileptic syndromes associated with NREM sleep, we recall Idiopathic Generalized Epilepsy (IGE, comprising a heterogeneous groups of epilepsies), Idiopathic Focal Childhood Epilepsies (IFCE), the Lennox-Gastaut Syndrome (LGS) and Temporolimbic Network Epilepsy (TLNE). Another major epileptic syndrome related to NREM sleep is nocturnal frontal lobe epilepsy (NFLE), whose features were originally defined during the detailed characterization of cohorts of patients affected by paroxysmal nocturnal dystonia (Provini et al., 1999; Tinuper and Lugaresi, 2002). The familial form of NFLE has autosomal dominant inheritance and was named ADNFLE (Scheffer et al., 1995). Because similar clinical and electroencephalographic (EEG) features are displayed by ADNFLE and the sporadic non-lesional cases of NFLE, ADNFLE is a good model of NFLE in general (Picard and Brodtkorb, 2008).

ADNFLE is a partial epilepsy often arising in childhood or early adolescence. Attacks arise in the frontal lobe, usually during stage 2 of sleep, and are characterized by clusters of complex and stereotyped hyperkinetic seizures. This suggests that motor patterns controlled by subcortical structures are released during the attacks. Sudden arousals are also frequent in ADNFLE, which is an indication of increased sleep fragmentation (Scheffer et al., 1995; Picard and Brodtkorb, 2008). Cognitive deficit and psychiatric comorbidities can also be present and are probably more common than initially assumed (Bertrand et al., 2005; Picard et al., 2009). As in many other epilepsies, about one third of the patients is refractory to pharmacological treatment. To date, hundreds of ADNFLE families have been identified. Nonetheless, because the genetic analysis is incomplete and because misdiagnosis is still frequent (Nobili et al., 2014), the exact incidence of the disease is unknown. Ten to fifteen percent of the ADNFLE families bear mutations on genes coding for subunits of the neuronal nicotinic receptor (Di Corcia et al., 2005; Picard and Brodtkorb, 2008; Ferini-Strambi et al., 2012; nAChR). The identification of other genes linked to ADNFLE has proceeded slowly. Recently, ADNFLE mutations have been detected in *KCNT1*, which codes for a Na^+^-gated K^+^ channel (Heron et al., 2012), and *DEPD5*, coding for the Disheveled, Egl-10 and Pleckstrin Domain-containing protein 5 (Ishida et al., 2013). Evidence is also available about the implication of the corticotropin-releasing hormone gene (*CRH*; Combi et al., 2005). Because little functional evidence is yet available for these genes, we here mostly focus on how mutant nAChRs may cause ADNFLE.

The nAChR is a pentameric ion channel permeable to cations, including Ca^2+^ (Fucile, 2004). When opened by ACh, it leads to membrane depolarization, which can produce post-synaptic excitation or, in presynaptic terminals, stimulation of neurotransmitter release. In the mammalian brain, nine subunits concur in forming functional nAChRs: α2-α7 and β 2-β 4, encoded respectively by the *CHRNA2-CHRNA7* and *CHRNB2-CHRNB4* genes. The two most common isoforms in thalamus and isocortex are the heteropentamer α4β 2^*^ and the homopentamer (α7)_5_ (Dani and Bertrand, 2007). The first ADNFLE mutation was identified in *CHRNA4* (Steinlein et al., 1995). To date, four mutations are known in *CHRNA4* and six in *CHRNB2* (Table 1), consistent with the major role of α4β 2^*^ nAChRs in regulating neocortical excitability (Wallace and Bertrand, 2013). The penetrance ranges from 60 to 80%. Some mutations are more frequently associated with psychiatric symptoms, but a specific relation between these symptoms and the functional alterations produced by the mutant subunits is not apparent (Steinlein et al., 2012). Finally, *CHRNA2* (Table 1) has also been causally associated with ADNFLE (Aridon et al., 2006). The α2 subunit can form heteromeric receptors by associating with both β 2 and β 4 (e.g., Hoda et al., 2009; Di Resta et al., 2010).

**Table 1:** **Mutations in ion channel-coding genes linked to ADNFLE**.

**Gene**	**Protein/subunit**	**Mutation**	**Reference**
*CHRNA4*	nAChR (α4)	S248F	Steinlein et al., 1995
		S252L	Hirose et al., 1999
		776ins3	Steinlein et al., 1997
		T265I	Leniger et al., 2003
*CHRNB2*	nAChR (β2)	V287L	De Fusco et al., 2000
		V287M	Phillips et al., 2001
		I312M	Bertrand et al., 2005
		L301V	Hoda et al., 2008
		V308A	
*CHRNA2*	nAChR (α2)	I279N	Aridon et al., 2006
*KCNT1*	Kca4.1	M896I	Heron et al., 2012
		R398Q	
		Y79 6H	
		R928C	

Besides the pathological relevance, the study of ADNFLE may offer important insight into the function of nAChRs in the cerebral cortex. In the present review, after summarizing some relevant morphofunctional features of the frontal lobe and its cholinergic innervation, we briefly discuss the sleep stages and their relation with ADNFLE and nAChRs. Next, we review the functional studies carried out with mutant nAChRs expressed in cellular systems or animal models. These studies are interpreted in the light of recent evidence about the physiological role of heteromeric nAChRs in different neocortical regions, in mature as well as developing brains. We next discuss the possibility of targeting the cholinergic system to treat ADNFLE. Finally, we briefly analyze the other genes that have been implicated in ADNFLE, as a perspective for future studies.

## The frontal lobes and the cholinergic system

In primates, the frontal lobes can be broadly divided into three functional regions: a caudal motor/premotor, a paralimbic (comprising the anterior cingulate complex, the parolfactory gyrus and the posterior orbitofrontal regions), and an extensive rostral heteromodal association area. The expression prefrontal cortex (PFC) is generally reserved to the latter two sections, which are extensively interconnected (Mesulam, 2000). The heteromodal PFC is also connected with the unimodal and heteromodal association cortices, while the paralimbic areas are directly linked to the limbic system. In this way, the emotional and visceral states are integrated with the sensory perception of external environment. The PFC is crucially implicated in cognitive tasks, and particularly in working memory, attention and goal-directed behavior. Extensive connections also exist between PFC and subcortical structures such as the basal nuclei (particularly the caudate nucleus) and the mediodorsal thalamus. PFC damage generally decreases the flexibility of behavior (Shallice and Burgess, 1991; Aron et al., 2004) and the capability of controlling impulse-driven responses (Mesulam, 2000). Importantly for the present perspective, prefrontal lesions in rodents produce similar alterations of executive functions (Dembrow and Johnston, 2014). Therefore, rodents seem to constitute a reasonable model to study several aspects of the frontal lobe physiology. For consistency with literature, we retain the expression PFC in rodents, although we are aware of the difficulties of carrying out comparisons with the much larger and complex dorsolateral PFC of primates (Uylings et al., 2003).

The frontal functions are regulated by ascending projections from hypothalamic and brain stem nuclei, which control the behavioral state and the sleep-waking cycle (Steriade and McCarley, 2005). In particular, the cerebral cholinergic system is composed of distinct groups of cholinergic neurons located in the pons and basal forebrain. These project diffusely to the brain, but especially to the cerebral cortex and thalamus (Mesulam, 2004). ACh cooperates with noradrenaline, histamine and serotonin to stimulate the neocortical tone, thus regulating arousal, attention and goal-directed behavior (Lucas-Meunier et al., 2003; Sarter et al., 2006; Wallace and Bertrand, 2013), as well as the switch between sleep stages (Saper et al., 2010). Work carried out in rodents indicates that the ascending cholinergic fibers densely innervate the somatosensory, visual, motor and prefrontal cortices, with a broadly homogeneous laminar distribution, especially in PFC (Eckenstein et al., 1988; Avendaño et al., 1996; Mechawar et al., 2000; Henny and Jones, 2008; Aracri et al., 2010).

The mechanisms by which ACh exerts its physiological functions are very complex. First, ACh can activate both ionotropic nAChRs and metabotropic (muscarinic) mAChRs. Early work tended to highlight the contribution of mAChRs, and the observed depolarizing effect of ACh on thalamic and neocortical neurons during wakefulness and REM sleep was initially attributed to a mAChR-dependent K^+^ channel inhibition (Krnjević et al., 1971; McCormick, 1992). More recently, the prominent role of nAChRs in regulating brain excitability and cognitive functions has been recognized (Picciotto et al., 1995, 1998; Hahn et al., 2003; Bailey et al., 2010; Howe et al., 2010; Guillem et al., 2011). However, the distinct roles of the numerous nAChR subunits expressed in the neocortex is poorly understood (Gotti et al., 2007; Pistillo et al., 2015). Second, although the overall effect of ACh in the neocortex is stimulatory, recent results indicate that the cholinergic terminals are often adjacent to the soma and dendrites of GABAergic interneurons, suggesting a strict relationship between the cholinergic and the GABAergic systems (Henny and Jones, 2008; Aracri et al., 2010). Therefore, alterations in cholinergic transmission may have subtle effects on the balance between excitation and inhibition in PFC circuits. Finally, several lines of evidence suggest that non-synaptic (paracrine) ACh release is widespread in the mammalian frontal cortex, but the balance of synaptic and non-synaptic effects of ACh is still matter of debate (Umbriaco et al., 1994; Mrzijak et al., 1995; Turrini et al., 2001; Descarries et al., 2004; Lendvai and Vizi, 2008; Aracri et al., 2010; Sarter et al., 2014).

## The stages of sleep and their relation with cholinergic transmission and epilepsy

Human sleep can be divided into several stages (Siegel, 2002; Steriade and McCarley, 2005). During attentive wakefulness, EEG mostly comprises low voltage (5–10 μV), high-frequency (20–40 Hz) *beta* waves. Ampler *alpha* oscillations at approximately 10 Hz appear on relaxed waking. On falling asleep, these are substituted by mixed EEG rhythms, with frequency band similar to the *beta*'s but higher amplitude (*stage 1* sleep). This brief phase is followed by *stage 2* sleep, in which the EEG displays *K-complexes* and *sleep spindles*, superimposed to the *beta* waves. The former are sharp, high voltage transient waves that may occur spontaneously or be triggered by sensory stimuli. The sleep spindles are wave discharges of 7–14 Hz, lasting 1–2 s, and recurring at 0.1–0.3 Hz with a waxing and waning pattern (Lüthi, 2014). During *stage 3* and *stage 4*, the EEG becomes progressively dominated by *delta* waves, with amplitudes up to 300 μV and a frequency of 0.5–3 Hz. These stages are collectively named *slow wave sleep* (SWS). Stage 1 to stage 4 constitute the NREM sleep, as indicated by electro-oculogram. From stage 4, the sleeper reverts to stage 3 and stage 2, from which the *rapid eye movement* (REM) state can be reached. In REM sleep, the EEG resembles the one observed during wakefulness, but the subject is unconscious and the skeletal muscle tone is minimum. The most vivid oneiric activity takes place in this stage. Sleep in non-human mammals is often simply described in terms of SWS and REM sleep, with durations shorter than in humans.

ACh release is high in wakefulness, strongly decreases during NREM sleep, and rises again in REM sleep (Jones, 2008). Many evidences point to the nicotinic receptors as important mediators of these effects. More specifically, studies with mice in which specific nAChR subunits were deleted show that the β 2^*^ nAChRs mediate most of the arousing effects of nicotine and regulate the NREM sleep stability. The transient activity of β 2^*^ nAChRs, caused by the low-level ACh release during NREM sleep, can stimulate “micro-arousal” events, at least in mice (Léna et al., 2004). In ADNFLE, this phenomenon could be potentiated by hyperfunctional α4β 2 nAChRs, thus causing the typical hyperexcitability with sudden arousals during NREM sleep. Such interpretation would also point to a strong implication of local neocortical cholinergic mechanisms in ADNFLE, which could explain an interesting neurophysiological difference between NFLE and most of the other sleep-related epilepsies (i.e., IGE, IFCE, LGS, and TLNE). In fact, the latter syndromes are characterized by EEG spike-wave discharges (Halász, 2013). These are thought to be caused by the interplay of cortical and thalamic cells. The “spike” reflects the strong activation of pyramidal cells, which is followed by the slower “wave” caused by the inhibition produced on these cells by the activation of local GABAergic interneurons as well as reticulothalamic (RT) cells. Spike-wave activity is facilitated during NREM sleep by the lower excitatory drive from the brain stem, which induces neuronal synchrony in the thalamocortical network (Amzica and Steriade, 2002; Halász, 2013). In contrast, in NFLE patients the spike-wave seizures are much less frequent (Halász, 2013), which points to an epileptogenic mechanisms characterized by a stronger implication of local neocortical circuits. This would be also in agreement with recent studies showing that cortical arousal is particularly sensitive to the excitatory input provided by the afferent fibers relayed through the basal forebrain, as compared to the thalamic afferents (Fuller et al., 2011 and references therein).

## The ADNFLE mutant nAChRs studied in expression systems

Most of the functional studies carried out to date concern the α4β 2 subtype, to which the mutant ADNFLE subunits confer a variety of functional alterations, whose physiological interpretation has given rise to controversy (Becchetti, 2012). Part of this diversity probably derives from the usage of different expression systems (*Xenopus* oocytes or mammalian cell lines), and of human or rodent nAChR subunit clones. Nonetheless, in the simulated heterozygous condition, which is the most relevant for rare and dominant mutations, a gain of receptor function is commonly observed. This is often caused by increased sensitivity to the agonists, accompanied or not by altered current kinetics (De Fusco et al., 2000; Phillips et al., 2001; Itier and Bertrand, 2002; Hoda et al., 2008). A similar increase was observed in the first mutation identified in the α2 subunit (Aridon et al., 2006; Hoda et al., 2009). One possible explanation of such an effect is that ADNFLE subunits modify the nAChR subunit ratio (Son et al., 2009). The α4β 2 receptor can exist in at least two stoichiometric forms: (α4)_3_(β2)_2_ and (α4)_2_(β 2)_3_. In heterologous expression systems, the former prevails and presents an EC_50_ for ACh of approximately 70 μM, whereas the latter subtype accounts for about 20% of the expressed receptors and presents an EC_50_ of around 1 μM (Nelson et al., 2003). In fact, five ADNFLE subunits (α4-S248F, α4-S252L, α4-776ins3, β 2-V287L, and β 2-V287M) were found to increase by 10–20% the proportion of the high-affinity subtype, when expressed in mouse neuroblastoma cells (Son et al., 2009). Another possible pathogenetic mechanism was suggested based on the nAChR response to extracellular Ca^2+^ ([Ca^2+^]_*o*_). The nAChRs are normally potentiated by increasing [Ca^2+^]_*o*_ up to the physiological concentration (Mulle et al., 1992; Vernino et al., 1992). Higher [Ca^2+^]_*o*_ produces channel inhibition (Buisson et al., 1996). The above five subunits, expressed in *Xenopus* oocytes, were observed to decrease the allosteric potentiation caused by [Ca^2+^]_*o*_ (Rodrigues-Pinguet et al., 2003). This could affect presynaptic α4β 2 receptors, leading to enhance glutamate release during synchronous discharges of pyramidal neurons (Rodrigues-Pinguet et al., 2003).

Regardless of the specific properties of individual mutations, it remains to be explained why mutant subunits widely expressed in the mammalian brain facilitate seizures in the frontal regions during light NREM sleep and why the attacks are accompanied by hyperkinetic motor events. Functional imaging studies show that complex alterations of heteromeric nAChR expression take place in human patients (Picard et al., 2006), which are difficult to explain on the basis of the functional alterations observed in cellular expression systems. Therefore, to better understand the effects of ADNFLE mutations in the complex cerebral context, it is necessary to proceed with broader studies of the cholinergic transmission in animal models of ADNFLE.

## Murine models of ADNFLE

Since 2006, several murine models of ADNFLE have become available. Klaassen et al. (2006) used C57BL/6J mice to generate knock-in strains expressing either α4-S252F or α4-+L264, respectively homologous to the human α4-S248F and α4-(776ins3). Heterozygous mice present recurrent seizures accompanied by increased nicotine-dependent GABA release in layer II/III of the PFC (Klaassen et al., 2006) and layer V of the motor cortex (Mann and Mody, 2008). On the other hand, in a different genetic background (CD1-129/Sv), expression of α4-S248F was found to confer a nicotine-induced dystonic arousal complex similar to the motor features of human ADNFLE, but no spontaneous seizures (Teper et al., 2007).

The other mutant subunit that has been widely studied in mice is β 2-V287L. A knock-in strain expressing β 2-V287L in C57BL/6 background displays a disturbed sleep pattern, abnormal excitability in response to nicotine, but no overt seizure phenotype (Xu et al., 2011; O'Neill et al., 2013). Moreover, conditional strains were generated expressing β2-V287L in FVB background, under control of the tetracycline promoter (TET-off system; Manfredi et al., 2009). Expression of β2-V287L does not alter the surface expression level of heteromeric nAChRs, but causes spontaneous seizures, generally during periods of increased *delta* wave activity. The epileptic phenotype is not reversed when β 2-V287L is silenced by administering doxycycline in adult mice. Conversely, when the transgene is silenced between the embryonic day 1 and the postnatal day 15, no seizures are observed, even if the transgene is expressed at a later stage.

Finally, in transgenic rats expressing α4-S284L, epileptic seizures are observed during SWS. These are accompanied by attenuation of synaptic and extrasynaptic GABAergic transmission before the onset of the epileptic phenotype and abnormal glutamate release at the onset of seizures (Zhu et al., 2008).

In summary, these murine models of ADNFLE do not display gross morphological alteration in their brains, but tend to display abnormal excitability, generally accompanied by disturbed sleep. The presence of spontaneous seizures is strain-dependent. The physiological analysis is incomplete, but points to altered GABAergic transmission in PFC. Furthermore, the only conditional model presently available suggests that critical stages of synaptic stabilization are implicated in the pathogenesis of ADNFLE.

## Nicotinic transmission in the cerebral cortex

### nAChRs in the PFC

As was mentioned earlier, the heteropentamer α4β 2^*^ and the homopentamer α7_5_ are the main nAChR subtypes in thalamus and isocortex (Gotti et al., 2007). Although the proportion of the different stoichiometries of α4β 2^*^ receptors *in vivo* are unknown, overall these receptors largely account for the highly sensitive and slowly desensitizing component of the response to ACh and nicotine. Therefore, the heteromeric nAChRs are thought to give a major contribution to the tonic control of neocortical excitability even in the presence of low ACh concentrations. In contrast, (α7)_5_ receptors have an EC_50_ for ACh of 100–200 μM, much faster desensitization and higher permeability to Ca^2+^ (Dani and Bertrand, 2007), and seem thus to be better suited to regulate the phasic responses to high ACh concentrations. This may explain why no ADNFLE mutation has ever been observed in *CHRNA7*, despite the widespread expression of α7 subunits in the brain.

The best characterized of the other subunits is α5, which can associate with α4β 2^*^ receptors (Kuryatov et al., 2008) and regulate its function in both mature and developing PFC (Ramirez-Latorre et al., 1996; Bailey et al., 2012). Unfortunately, nothing is known about the interaction of α5 with the known ADNFLE subunits. Very little is also known about the physiology of α2, which is directly implicated in ADNFLE (Aridon et al., 2006). The difficulty of interpreting the mutations on α2 resides in the fact that its pattern of expression is different in primates as compared to rodents. In particular, α2 is much more widely expressed in primates' brain (Han et al., 2000; Aridon et al., 2006), where there seems to be much more overlap with α4 than in rodents' brain. The other complication is that α2, as α4, can associate with both β 2 and β 4 to yield functional nAChRs. The cerebral expression of β 4 is also relatively widespread in mouse (Gahring et al., 2004), squirrel monkeys (Quik et al., 2000) and human *feti* as well as aged post-mortem samples (Hellström-Lindahl et al., 1998). However, once again, scarce attention has been devoted to the physiology of β 4. Nevertheless, the above evidences suggest that heteromeric receptors different from α4β 2 could have important physiological roles in the mammalian brain, and particularly in primates.

### nAChRs regulate both excitatory and inhibitory transmission

In general, β 2^*^ nAChRs regulate glutamate release from thalamocortical fibers (Vidal and Changeux, 1993; Gioanni et al., 1999; Lambe et al., 2003). The expression and roles of nAChRs on pyramidal cell somata and terminals are more variable, depending on species, layer and cerebral region (Chu et al., 2000; Couey et al., 2007; Dickinson et al., 2008; Kassam et al., 2008; Zolles et al., 2009; Marchi and Grilli, 2010; Poorthuis et al., 2012; Aracri et al., 2013a). Nonetheless, the global effect of ACh release in deep layers is thought to be excitatory and dominated by β 2^*^ receptors (Poorthuis et al., 2012). This is relevant in the present context, as layer V is particularly prone to develop seizures (Richardson et al., 2008). During NREM sleep, the cholinergic tone is low, but hyperfunctional nAChRs could maintain abnormal glutamate release, even in the face of low ACh levels, thus increasing sleep fragmentation. In fact, the rat strain expressing α4-S284L shows higher nicotine-dependent glutamate release during SWS (Zhu et al., 2008). It seems however unlikely that hyperfunctional nAChRs can produce paroxystic hyperexcitability by a moderate stimulation of glutamate release in the neocortex. Because the pyramidal neuron activity is potently regulated by the feed-back control exerted by GABAergic neurons, some degree of circuit disinhibition is generally required to lead to seizure-like activity (Richardson et al., 2008). This is confirmed by theoretical modeling showing that altering the “weight” of inhibitory connections is the most effective way of modulating the excitability of recurrent networks (Tsodyks et al., 1997; Murphy and Miller, 2009; Ozeki et al., 2009). Therefore, to understand the nAChR-dependent hyperexcitability it is necessary to also consider the nicotinic regulation of neocortical GABAergic transmission. In fact, expression of heteromeric nAChRs on the soma of different interneuronal populations is established in rats (Xiang et al., 1998; Porter et al., 1999; Christophe et al., 2002), humans (Alkondon et al., 2000) and mice (Couey et al., 2007; Aracri et al., 2010). Heteromeric nAChRs also exert presynaptic control of GABA release onto pyramidal cells (Klaassen et al., 2006; Aracri et al., 2010).

## The origin of seizures in ADNFLE

**Figure 1:**
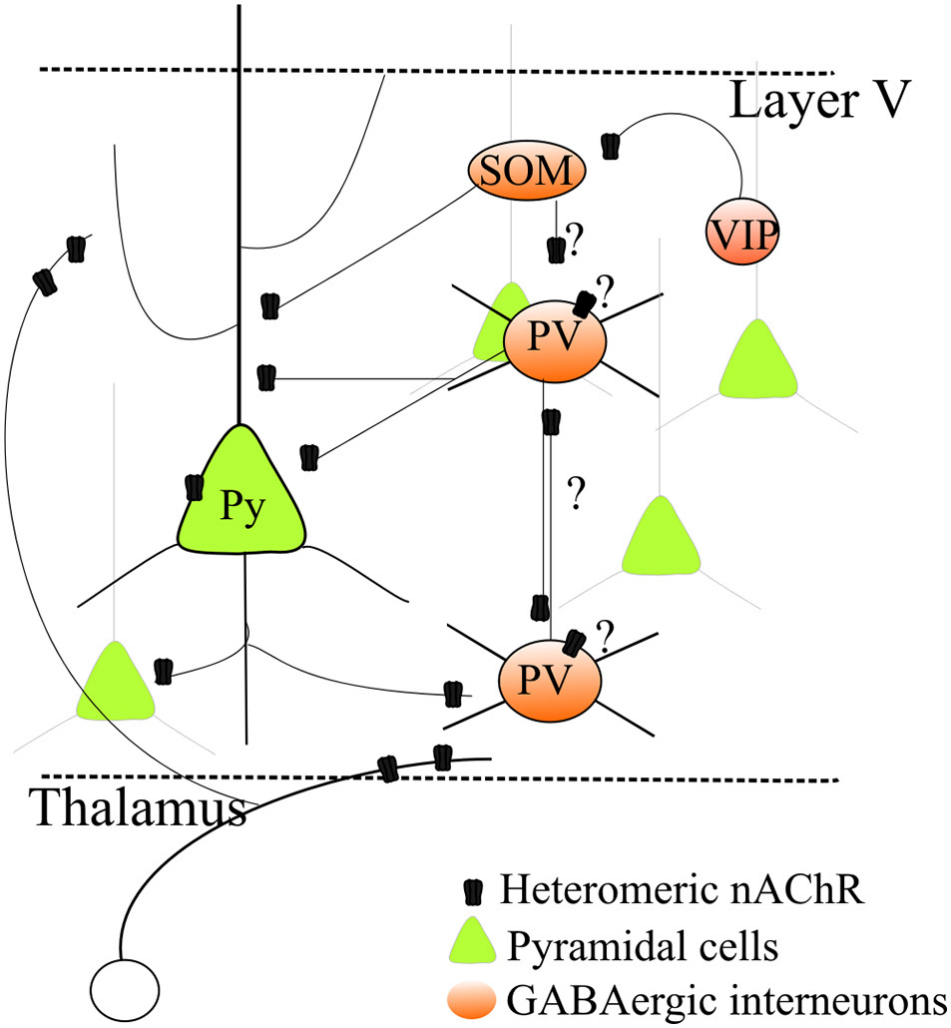
**Regulation by heteromeric nAChRs of a simplified layer V neocortical microcircuit**. Heteromeric β 2^*^ nAChRs regulate excitatory transmission by stimulating glutamate release from thalamocortical as well as intrinsic glutamatergic terminals. Expression of nAChRs on pyramidal cell somata has also been observed, although it is more variable. Heteromeric nAChRs also control GABA release onto pyramidal neurons. Moreover, growing evidence indicates that nAChRs are also expressed in several distinct types of GABAergic cells. However, because their precise physiological roles in the different cell types is unclear, a comprehensive picture cannot be given yet. This uncertainty is indicated by the question marks in the graph. For full discussion and references, see the main text.

In the light of the above discussion, ADNFLE seizures could be triggered by several mechanisms. (1) During SWS, thalamocortical neurons tend to be inhibited (Steriade and McCarley, 2005). In these conditions, an upsurge of ACh in the presence of hyperfunctional nAChRs could cause excessive GABA release in the PFC and thus abnormal hyperpolarization of pyramidal neurons. This would deinactivate low-threshold, voltage-gated Ca^2+^ channels and activate pacemaker H-type currents, thus making pyramidal cells more sensitive to post-inhibitory rebound (Klaassen et al., 2006). (2) Alternatively, nAChR activation could stimulate reciprocal inhibition between GABAergic interneurons, thus producing pyramidal cell disinhibition (Figure 1). This latter mechanism has been excluded in Klaassen's work (2006), but may explain Zhu's results (2008). (3) Another possible mechanism considers thalamocortical interplay. In stage 2 of sleep, spindle waves are generated in the thalamus by the regulatory action of RT cells onto thalamocortical cells (Lüthi, 2014), but cortical neurons are essential in synchronizing their appearance in wide thalamocortical regions. Sleep spindles can turn into epileptiform activity and could be promoted by nAChR-dependent stimulation of glutamate release onto RT cells and the release of GABA from RT cells onto thalamocortical cells (Sutor and Zolles, 2001).

A more detailed interpretation is made difficult because of the current uncertainties about the GABAergic cell populations and their physiological roles in the neocortex. The classification of GABAergic interneurons must take into account a number of factors, such as morphology, axonal and dendritic connectivity, efficacy and dynamics of input and output synapses, intrinsic electrophysiological properties, combinations of molecular markers, such as Ca^2+^-binding proteins and neuropeptides, and developmental origin (Markram et al., 2004; DeFelipe et al., 2013; Cauli et al., 2014). The role of different GABAergic populations in the cortical microcircuit is unclear, and differences occur between layers, brain areas and species. A simplified classification (Figure 1), which covers more than 85% of cortical interneurons, is based on expression of parvalbumin (PV), somatostatin (SOM) and vasoactive intestinal peptide (Rudy et al., 2011; Kepecs and Fishell, 2014; VIP). PV neurons are generally fast-spiking (FS) cells (Hu et al., 2014), which are thought to be the main responsible of surround inhibition, particularly in layer V. Models of interactions between the GABAergic populations were proposed based on studies on hippocampus and sensory, but not associative, cortices (Pfeffer et al., 2013). While PV-cells mainly inhibit the perisomatic compartments of pyramidal cells, SOM-cells form synapses onto dendrites of pyramidal neurons and inhibit PV interneurons (but not *vice versa*). SOM-cells would thus not only increase inhibition in the dendrites but also decrease the perisomatic inhibition mediated by PV-cells (Pfeffer et al., 2013). In contrast, VIP-cells produce scarce inhibition of pyramidal cells, but seem to specifically target SOM-cells. In this way, VIP-cells may indirectly stimulate PV-cells. This simplified picture is complicated by the presence of chandelier cells, a subtype of PV-cells that mainly targets the axon hillock of pyramidal cells. Their function seems critical in PFC (Hardwick et al., 2005) and shows species-specific characteristics possibly related to neurologic disorders (Woodruff and Yuste, 2008).

The study of how nAChRs regulate GABAergic populations is in its infancy (Figure 1). Nonetheless, the non-FS cells (mainly SOM, probably) that form GABAergic synapses onto FS-PV cells (Harris and Mrsic-Flogel, 2013; Pfeffer et al., 2013) express α7, α4, and β 2 nAChR subunits (Freund, 2003; Couey et al., 2007; Armstrong and Soltesz, 2012). Therefore, mechanism (2) above could be facilitated in SWS, when glutamatergic transmission weakens. In these conditions, inhibition between interneurons may prevail because of nicotinic excitation of the cell bodies of the non-FS cells that innervate the FS-PV-cells. Moreover, FS-cells also present reciprocal inhibitory connections (Gibson et al., 1999; Galarreta and Hestrin, 2002). Because FS-cell terminals express α4β 2 nAChRs, it is possible that when the overall glutamatergic input decreases, the action of hyperfunctional nAChRs on presynaptic terminals shifts the synaptic balance toward interneuronal inhibition.

## Why do seizures arise in the frontal regions?

Besides the local mechanism by which ADNFLE mutations can alter the excitability of neocortical circuits, one must also explain why seizures arise in frontal regions. Although the literature on cholinergic transmission is immense, the analysis of the differences in cholinergic and, in particular, nicotinic regulation of different areas of the isocortex is rather neglected. Interestingly, recent results suggest that important differences exist in cholinergic innervation and response to nicotine between distinct regions of the cerebral cortex. In mouse, for instance, the response of layer VI pyramidal neurons to nicotine in the medial PFC is more sensitive than the one observed in primary somatosensory and motor cortices (Tian et al., 2014). This appears to be consistent with the distribution of cholinergic fibers in infragranular layers, which is denser in PFC, as compared to primary sensory and motor regions (Aracri et al., 2010). Differences in cytoarchitectonics and physiology are also observed between different prefrontal regions (for a brief discussion, see Aracri et al., 2013a).

Moreover, a typical feature of ADNFLE is the presence of stereotyped motor events accompanying the seizures, suggesting that motor patterns are released during the attacks. Therefore, we hypothesize that the implication of premotor areas such as Fr2 (also known as M2; discussed in Aracri et al., 2013a) is particularly relevant for ADNFLE. This area projects to the motor cortex and dorsolateral striatum (Berendse et al., 1992; Condé et al., 1995). Moreover, it is very sensitive to nicotinic stimulation in layer V (Aracri et al., 2010, 2013a), and its well-developed layer V is seemingly accompanied by lesser inhibitory weight than is typical in regions such as the somatosensory cortex (Aracri et al., 2013a).

## Developmental aspects

The subtle prefrontal circuit alterations that cause ADNFLE seizures are likely to be produced during the developmental phases of network stabilization, as is also indicated by conditional expression of β 2-V287L (Manfredi et al., 2009). In mammals, a “brain growth spurt” occurs around birth, characterized by neurite outgrowth, synaptogenesis, myelinization and circuit pruning (Eriksson et al., 2000). In rodents, this phase spans the first 3, 4 postnatal weeks and is accompanied by maturation of the cholinergic system and an upsurge of nAChR expression. In rat forebrain, β 2 appears in mid-gestation and peaks in the second postnatal week, along with α4 and α7 (Mansvelder and Role, 2006). A similar pattern is observed in mouse (Kassam et al., 2008; Bailey et al., 2012), although evidence is less extensive. At this stage, the density of extrinsic cholinergic innervation (Mechawar et al., 2002) and cortical cholinergic cells (Consonni et al., 2009) increases dramatically. Treatment with nicotinic ligands in the second postnatal week produces persistent behavioral and morphological alterations (Eriksson et al., 2000) and mice lacking β 2 show region-specific changes in cortical structure (Cordero-Erausquin et al., 2000). Nonetheless, the specific nAChR roles during neural circuit wiring are still largely unknown. The spontaneous nAChR activity was reported to regulate the developmental switch between the excitatory and inhibitory roles of GABA (Liu et al., 2006). The latter transition depends on the progressive substitution of the transporter NKCC1, which absorbs Cl^−^ and is mainly expressed in early stages, with KCC2, which extrudes Cl^−^ and is expressed at later stages (Rivera et al., 1999; Ben-Ari et al., 2012). Both homo- and heteromeric nAChR activity seem to regulate expression of these transporters (Liu et al., 2006). In particular, KCC2 appears after postnatal day 3 in layer V pyramidal neurons. Its expression accompanies the formation of GABAergic synapses and KCC2 variants have been associated to epilepsy (Kaila et al., 2014; Puskarjov et al., 2014). Precise timing of early GABAergic excitation is important for early neuronal development and integration into circuits (Ge et al., 2006; Ben-Ari et al., 2012). Recent work in transgenic rats expressing α4-S284L (Zhu et al., 2008) suggest that this mechanism may also be active at a later stage. In these mice, the onset of the epileptic seizures (around 8 weeks after birth) is accompanied by a decrease of the expression ratio of KCC2 and NKCC1, with a consequent depolarizing shift of the GABA_*A*_ reversal potential, which could explain the observed alterations in GABAergic transmission (Yamada et al., 2013).

## Pharmacological treatment of ADNFLE

A first-line choice for treating ADNFLE is carbamazepine (CBZ). As many other antiepileptics (AEDs), CBZ has inhibitory effects on voltage-gated Na^+^ channels (McLean and Macdonald, 1986). Nonetheless, CBZ has been proposed to be particularly effective on ADNFLE because it also blocks heteromeric nAChRs at therapeutic doses, and ADNFLE mutations alter the channel sensitivity to the drug (Picard et al., 1999; Hoda et al., 2009). However, CBZ can cause serious toxic side-effects produced by its metabolites. A related less toxic second generation compound is oxcarbazepine (Beydoun et al., 2008), which has recently found to give good results in ADNFLE patients, including some refractory to other drugs (Raju et al., 2007; Romigi et al., 2008). The steady-state plasma concentration of oxcarbazepine is negligible and the clinically relevant compound is thought to be the monohydroxy derivative MHD, which can reach effective plasma levels between 30 and 150 μM (Johannessen et al., 2003). In agreement with the notion that good efficacy in treating ADNFLE may depend on modulation of nAChRs, oxcarbazepine and MHD can also inhibit heteromeric nAChRs, especially α4β 2 (Di Resta et al., 2010). Lamotrigine, another AED commonly used in partial epilepsy (Labiner et al., 2009), has also been found to block α4β 2 nAChRs (Zheng et al., 2010). Unfortunately, the effects of common AEDs on ligand-gated ion channels have begun to be studied only recently (Di Resta and Becchetti, 2013). Deeper analyses on the action of AEDs on different ion channels and the search of more specific compounds are clearly needed to advance the pharmacology of ADNFLE and epilepsy in general.

Considering that many ADNFLE mutations produce an overall increase of receptor's function and that several AEDs block nAChRs, it may seem paradoxical that treatment with nicotine has also been suggested to be beneficial in ADNFLE. After the first report in a patient refractory to standard antiepileptic therapy (Willoughby et al., 2003), the effect of tobacco habit was studied in a wider cohort of patients carrying either α4-776ins3 or α4-S248F (Brodtkorb and Picard, 2006). Seizure freedom was associated with smoking habits and transdermal nicotine application had beneficial effects in one patient (Brodtkorb and Picard, 2006). The possible explanations of these observations are as follows. First, tonic nicotine administration tends to produce partial nAChR desensitization, which may counteract the effects of receptors' hyperfunctionality. Second, nicotine can act as a molecular chaperon that regulates the nAChR subunit expression (Kuryatov et al., 2005). In several ADNFLE mutations studied *in vitro*, tonic nicotine application was observed to decrease the overexpression of the high-affinity (α4)_2_(β 2)_3_ subtype (Son et al., 2009). Therefore, in ADNFLE patients, nicotine may normalize an altered subunit stoichiomentry.

Investigating the pathogenesis and pharmacology of epilepsy is complicated by the scarcity of good *in vitro* models, as it is difficult to obtain spontaneous epileptiform activity in cultured neuronal networks or brain slices. In the case of ADNFLE, long-term neuronal cultures dissociated from mice expressing β 2-V287L display spontaneous hyperexcitability features (i.e., not requiring the application of pro-convulsants), as measured with multi-electrode arrays (Gullo et al., 2014). The network excitability can be modulated by both GABAergic drugs and CBZ. Besides facilitating the study of the role of β 2-V287L on synaptic formation, this experimental model allows to determine the effects of tonic pharmacological treatment on network excitability. In general, *in vitro* models such as this one should considerably facilitate the screening of antiepileptic drugs (AEDs).

## Other genes implicated in ADNFLE

### KCNT1

Four missense mutations in the *KCNT1* gene (Table 1), coding for a Na^+^-gated K^+^ channel (KCNT1 or K_Ca_4.1, also known as Slo2.2 or Slack), were associated to severe ADNFLE with psychiatric symptoms, and a penetrance of 100% (Heron et al., 2012). Consistently, KCNT1 is expressed in the frontal region (Bhattacharjee et al., 2002). The ADNFLE mutations tend to cluster around the cytoplasmic NAD^+^ binding domain (Heron et al., 2012), which regulates the channel sensitivity to [Na^+^]_i_ (Tamsett et al., 2009). When expressed in *Xenopus laevis* oocytes, these mutations produce higher currents than the WT counterparts. In analogy with what was discussed for the mutant nAChRs, the balance of the possible physiological effects on excitatory and inhibitory transmission is uncertain. It is possible that hyperfunctional KCNT1 channels accelerate the action potential repolarization, thus increases the firing frequency of pyramidal neurons (Milligan et al., 2014). Because KCNT1 is also expressed in interneurons (Bhattacharjee et al., 2002) it has been also suggested that the slow accumulation of Na^+^ in FS interneurons could stimulate KCNT1 to dampen excitability. The effect would be stronger in the case of mutant channels, with ensuing network disinhibition (Milligan et al., 2014).

### DEPD5

Loss-of-function mutations in *DEPDC5* are linked to different types of focal epilepsies, including ADNFLE (Dibbens et al., 2013; Ishida et al., 2013; Lal et al., 2014; Picard et al., 2014; Scheffer et al., 2014). Differently from the mutations on ion channel genes, the epileptogenic mutations in *DEPDC5* are unrelated to the specific area of seizure initiation. Therefore, *DEPDC5* appears to be implicated in overall excitability, rather than being specifically associated with ADNFLE. The DEPDC protein participates in a molecular complex implicated in repressing the activity of mTORC1 (Target Of Rapamycin Complex 1; Bar-Peled et al., 2013). However, how the mTORC1 pathway may be linked to epileptogenesis is unknown (Baulac, 2014).

### The possible involvement of neuropeptides

Putative disease-causing mutations have been identified in the promoter (Combi et al., 2005) or in the pro-sequence region of *CRH* (Combi et al., 2005; Sansoni et al., 2013). *In vitro*, the P30R substitution in the pro-sequence of CRH decreases peptide secretion (Sansoni et al., 2013). How suppressed CRH signaling may lead to ADNFLE seizures is unclear. Among neuropeptides, CRH is known to exert the most potent pro-epileptogenic effects during development (Baram and Hatalski, 1998). The hypothalamic neurons secreting CRH also innervate the neocortex, and CRH receptors are densely distributed in rodents' prefrontal regions (Radulovic et al., 1998; Van Pett et al., 2000), thus cooperating with the ascending arousal systems, including the cholinergic, particularly during the stress response. The other hypothalamic neuropeptide implicated in neocortex arousal and sleep-wake cycle is orexin (also known as hypocretin). Orexin peptides directly regulate synaptic transmission in the frontal cortex (Li et al., 2010; Aracri et al., 2013b), and cooperate with ACh in modulating glutamate release from thalamocortical fibers (Lambe et al., 2005). Until now no ADNFLE-linked mutations have been observed on orexin-related genes (Bouchardy et al., 2011). Nonetheless, we believe that the interplay between ACh, orexin and CRH in regulation nurotransmission in the cerebral cortex may be another fruitful line of research with implications for sleep-related epilepsy in general and ADNFLE in particular.

## Conclusions

Several genes have been associated with ADNFLE, but the available functional studies mostly concern heteromeric nAChRs. Murine models suggest that alteration in the nicotinic control of GABAergic transmission may be a major pathogenetic mechanism. In general, the prefrontal regions may be particularly sensitive to nicotinic stimulation in the deep layers. However, a full comprehension of epileptogenesis in ADNFLE will require a better understanding of how the different nAChR subtypes regulate both excitatory and inhibitory neurons, at pre- and postsynaptic level, in the frontal regions. In particular, how nAChRs control different interneuronal populations is unclear. Moreover, assessing how the altered excitability is generated during the maturation of neocortical connections will need deeper studies of the nAChR function in the early postnatal weeks. From a pharmacological standpoint, mounting evidence indicates that several classic AEDs can target neurotransmitter-gated ion channels, and particularly nAChRs. Modulating nAChRs is a possible therapeutic strategy in ADNFLE, which merits further investigation.

### Conflict of interest statement

The authors declare that the research was conducted in the absence of any commercial or financial relationships that could be construed as a potential conflict of interest.
